# ASO Author Reflections: Expanding Access to Hepatic Artery Infusion Therapy: A Feasible Port-Based Model in a Global Context

**DOI:** 10.1245/s10434-025-18168-x

**Published:** 2025-08-26

**Authors:** Roy Apel, Eran Sadot

**Affiliations:** 1 Department of Surgery, Rabin Medical Center, Beilinson Hospital, Petach Tikva, Israel; 2 Tel Aviv University, Gray Faculty of Medicine, Tel Aviv, Israel

## Past

Hepatic artery infusion (HAI) therapy has demonstrated clinical benefit in patients with colorectal liver metastases (CRLM), particularly in select centers in North America where Floxuridine (FUDR)-based HAI is delivered via implantable pumps.^1^ However, this approach is often limited by cost, complexity, and regional availability. In many parts of the world, including Israel, HAI therapy had not been accessible.^2^ The challenge was to develop a safe, effective, and more accessible alternative to pump-based HAI, particularly one compatible with commonly available chemotherapy agents and healthcare infrastructure.

## Present

This study reports the first clinical experience of a port-based HAI program for CRLM in Israel.^3^ Using a reservoir-less port system and oxaliplatin or mitomycin-based regimens, we demonstrate that this approach is both feasible and safe. The treatment was successfully delivered with a low complication rate, a substantial conversion-to-resection rate among patients treated with neoadjuvant intent, and encouraging short-term oncologic outcomes. These findings highlight the potential of the port-based approach, which is cost-effective both in terms of device and drug costs and avoids the use of Freon gas, which is prohibited in some countries for environmental reasons.^2^ These findings underscore the potential of adapting HAI therapy to different healthcare settings by leveraging affordable and accessible alternatives to the pump-based systems.

## Future

Further research is needed to evaluate the long-term oncologic outcomes and late complications of port-based HAI. Comparative studies between pump-based and port-based strategies may help define the optimal settings and patient populations for each. Ultimately, the goal is to expand access to HAI therapy worldwide by adapting it to diverse healthcare systems and enhancing multidisciplinary collaboration in the care of patients with CRLM.
